# Focus on gut microbiota regulation: exploring the potential of fermented traditional Chinese medicines in the prevention and treatment of type 2 diabetes mellitus

**DOI:** 10.3389/fmicb.2026.1763653

**Published:** 2026-02-11

**Authors:** Ruodi Yang, Yufeng Yang, Ying Zhou, Yuhang Shen, Yan Shi, Juntong Liu

**Affiliations:** 1 Department of First Clinical School, Liaoning University of Traditional Chinese Medicine, Shenyang, China; 2 Department of Academic Affairs Office, Liaoning University of Traditional Chinese Medicine, Shenyang, China; 3 Department of Basic Medical Sciences, Liaoning University of Traditional Chinese Medicine, Shenyang, China; 4 Department of Teaching and Experiment Center, Liaoning University of Traditional Chinese Medicine, Shenyang, China

**Keywords:** gut microbiota, novel technology, short-chain fatty acid, traditional Chinese medicine fermentation, type 2 diabetes mellitus

## Abstract

Type 2 diabetes mellitus (T2DM), a globally prevalent metabolic disorder, has dysbiosis of the gut microbiota as a significant pathogenic factor. Traditional Chinese medicine (TCM) fermentation, originating from traditional processing techniques, is a technology that combines modern microbiological methods with solid-state fermentation, submerged fermentation, and bidirectional fermentation of medicinal fungi. Under specific conditions, it facilitates the biotransformation of herbal raw materials, demonstrating distinct advantages in regulating gut microbiota. This study aims to outline the concept of fermented TCM, elucidate the relationship between gut microbiota and T2DM, and explore the mechanisms by which fermented TCM modulates gut microbiota to improve T2DM. Literature searches in databases such as PubMed, Google Scholar, and Web of Science reveal that fermented TCM improves T2DM by targeting gut microbiota regulation as a core mechanism. The mechanisms may involve: modulating gut microbiota composition (fermentation products increase beneficial bacteria abundance, decrease harmful bacteria proportion, and restore microbial balance); influencing gut microbiota metabolites (promoting short-chain fatty acid (SCFA) production by microbiota, which participates in glucose and energy metabolism); protecting intestinal barrier function (SCFAs enhance intestinal epithelial cell function, upregulate tight junction protein expression, preserve barrier integrity, and reduce endotoxin leakage into the bloodstream); and modulating intestinal immune function (inhibiting inflammatory responses, enhancing antioxidant activity, and regulating intestinal immune homeostasis). This study reviews the application research of fermented TCM in improving T2DM by regulating the gut microbiota, aiming to validate and reveal its potential in the prevention and treatment of T2DM through gut microbiota modulation.

## 1 Introduction

Diabetes mellitus (DM) is a prevalent metabolic disease worldwide characterized by chronic hyperglycemia. Type 2 diabetes mellitus (T2DM) is the largest and most common type of diabetes and develops primarily as a result of insulin resistance and a relative lack of insulin secretion (Singh et al., 2025). Data show that T2DM accounted for 96.0% of all global diabetes cases in 2021, and projections indicate that by 2050, 1.31 billion people will live with diabetes worldwide (Ong et al., 2023). Prolonged hyperglycemia can lead to complications in various organ systems, including the heart, brain, kidneys, and peripheral nerves, significantly impacting patients' quality of life (Dilworth et al., 2024). Globally, T2DM has become a serious public health issue (Singh et al., 2025). It has therefore become crucial to actively explore the pathomechanisms of T2DM and the effective methods for combating it.

In recent years, the gut microbiota has been widely recognized in academic circles as a novel key target for combating T2DM (Liu et al., 2022). Diet, as the primary external factor regulating the intestinal microecology, plays an irreplaceable role in shaping the community composition, metabolic function, and species diversity of gut microorganisms. Different dietary habits directly influence the structural stability of the gut microbiota, while also exerting profound effects on its functional activity and species richness (Ross et al., 2024). Research indicates that the persistent maintenance of poor dietary habits, such as high sugar and fat intake, significantly disrupts the equilibrium of the gut microbiota, leading to dysbiosis. This disruption in microbial structure and function constitutes a critical pathological link in the onset and progression of T2DM (Hills et al., 2019). It is thus evident that gut microbiota dysbiosis has emerged as a key trigger in the pathogenesis of T2DM. Effectively regulating gut microbiota balance shows promise as a crucial intervention point for preventing the onset of T2DM and slowing its progression.

Currently, numerous hypoglycemic agents exert significant effects in the treatment of T2DM by indirectly modulating the gut microbiota. However, the lowering of blood glucose is often accompanied by certain side effects or adverse reactions. Metformin, as a frontline drug treatment for T2DM, exerts its hypoglycemic effects not only by inhibiting hepatic glucose output and enhancing insulin sensitivity in peripheral tissues, but also through the crucial indirect pathway of modulating the gut microbiota (Sun et al., 2018; Weersma et al., 2020). It is worth noting, however, that gastrointestinal side effects, such as diarrhea, bloating, and nausea, occur in about one-third of the patients who take metformin (McCreight et al., 2016). About 5% of the patients cannot even tolerate metformin (Dujic et al., 2015). Glucagon-like peptide-1 receptor agonists (GLP-1RAs) are commonly used medications for the treatment of T2DM. Research indicates that gut microbiota modulation is also one of the key mechanisms underpinning their enhanced efficacy. However, some patients still experience adverse reactions such as nausea, constipation, and diarrhea following administration, which are considered outcomes of the pharmacological action of GLP-1RAs (Kato et al., 2021). Acarbose is widely used in postprandial blood glucose management. However, research reveals that its efficacy is diminished through degradation by specific gut microbiota, placing a significant proportion of the population at risk of developing resistance (Tian et al., 2023).

Fermentation technology is an ancient production method derived from the development of human civilization. After thousands of years of development, fermentation technology in traditional Chinese medicine (TCM) has evolved into a widespread and crucial processing technique for Chinese herbal medicines. Modern research on fermented TCM has gradually become an active field in the modernization of TCM, offering new avenues for R&D in the prevention and control of various diseases (Zhang et al., 2012). Directed fermentation of TCM using a single strain or a mixture of strains can alter the drug's properties, enhance its efficacy, and even reduce its toxicity, side effects, and adverse reactions. Nevertheless, the mechanism of TCM fermentation still lacks clarity, and the interaction mechanism between active ingredients, inactive ingredients, other special substrates, and microorganisms remains poorly understood (Li et al., 2020). Research has demonstrated that fermented TCM exerts a regulatory effect on the composition of broiler chicken gut microbiota, altering their original flora structure (Huang et al., 2021). This finding provides novel targets for investigating the mechanisms underlying fermented TCM. In particular, probiotic strain combinations in fermented TCM are of great significance for maintaining intestinal microecology (Ma et al., 2025). Multiple studies have also confirmed that fermented TCM can effectively enrich beneficial gut microbiota, inhibit the proliferation of harmful bacteria, and simultaneously maintain the diversity and structural stability of the intestinal microbiome (Duan et al., 2024; Li et al., 2025). Furthermore, through microbial fermentation, these preparations effectively reduce the inherent properties of raw herbs, enhance the bioavailability of herbal components, significantly decrease gastrointestinal irritation, and markedly improve patient tolerance (Fan et al., 2025). Thus, fermented TCM demonstrates significant advantages in long-term regulation of gut microbiota for preventing and treating T2DM, offering a novel approach to address the challenges of side effects and adverse reactions associated with traditional hypoglycemic drugs. Based on this, the study will take the gut microbiota as a bridge to establish a link between the fermentation technology of TCM and the combating of T2DM. It will also elaborate on the potential mechanisms by which TCM fermentation technology regulates the gut microbiota, as well as research regarding its application in T2DM prevention and treatment, thereby providing a novel technological reference for the combating of T2DM by using Chinese herbal medicine.

## 2 Overview of fermentation in TCM

### 2.1 The concept of TCM fermentation technology

Fermentation of TCM is a process that integrates traditional TCM processing theory with modern microbial fermentation technology. It involves the biological transformation of Chinese medicinal raw materials by utilizing specific microorganisms or their metabolites under controlled environmental conditions, such as temperature, pH, and humidity (Li et al., 2020). It originates from ancient traditional fermentation practices, which were historically applied to the fermentation of meats, wines, and dairy products (Cavalieri et al., 2003; Leroy et al., 2023; Zheng et al., 2022). During the Eastern Han Dynasty, Zhang Zhongjing described fermented Chinese medicinal substances such as Massa Medicata Fermentata in his Synopsis of the Golden Chamber. By this period, fermentation techniques in TCM had begun to take shape. Among the formulas recorded in the Treatise on Cold Damage, the application of the *Zhizichi* Decoction stands as a landmark example of fermentation technology finding its initial application within the realm of formulaic medicine. With the advancement of modern biotechnology, TCM fermentation technology has made significant progress with the help of modern technologies such as microbial technology, fermentation engineering, and bioengineering. TCM fermentation is usually categorized into three types according to the fermentation method: solid-state fermentation, submerged fermentation, and bidirectional fermentation of medicinal fungi in TCM (Zhang X. et al., 2023).

### 2.2 Types of TCM fermentation

#### 2.2.1 Solid-state fermentation (SSF)

SSF of TCM is a process in which microorganisms grow, reproduce, and metabolize to produce target products in an environment with a lack of free water or a very low content of free water, using solid substrates such as Chinese herbal powder as carriers (Ikusika et al., 2024). The substrates employed are predominantly water-insoluble polymeric substances. Such substrates not only provide functional microorganisms with the carbon sources, nitrogen sources, inorganic salts, moisture, and other essential materials required for growth, but also serve as platforms that facilitate microbial growth and reproduction. SSF is extensively utilized in the food and beverage industry, and is commonly used in the manufacturing of vinegar, white wine, soy sauce, and other products (Jin et al., 2024). Advances in scientific research and technology have driven the development and application of SSF technology. Its biological processes are also widely used in the remediation and degradation of hazardous substances, the detoxification of agricultural and industrial wastes, enhancing crop and residue nutrition, the mass production of secondary metabolites (e.g., antibiotics), and the production of enzymes, organic acids, biopesticides, and biopharmaceuticals (Chilakamarry et al., 2022). The SSF of TCM is often applied in cases where the fermentation conditions are not demanding, and where the original form of the TCM needs to be preserved. The core advantage of SSF lies in the strong adaptability of raw materials, which can efficiently utilize various types of low-cost, complex components of the substrate, especially suitable for processing “high-fiber, high-solids content” raw materials, and significantly increase the value of resources. At the same time, the low-moisture environment of solid substrates can simulate the natural growth habitats of microorganisms, reducing fermentation costs, energy consumption, and wastewater discharge (Krishna, 2005).

#### 2.2.2 Submerged fermentation (SmF)

SmF, also referred to as liquid-submerged fermentation, entails the processing of Chinese medicinal raw materials in a liquid medium. Under suitable conditions such as temperature and pH, microorganisms grow in a liquid environment rather than a solid one. Compared with SSF, SmF offers the advantages of high product stability, quantifiable production conditions, and a high level of automation. It is therefore a highly effective application in the large-scale fermentation of TCM (Li et al., 2020). However, this also makes the technical requirements of SmF extremely strict, as it requires a strict sterilization environment. In particular, the fermentation equipment and conditions must be precisely controlled to maximize the conversion rate of active ingredients while minimizing the risk of contamination. Most probiotics such as *Lactobacillus* and *Bifidobacterium* are partially anaerobic or anaerobic microorganisms, and it is easier to achieve precise control of the fermentation parameters in a liquid environment so that the activity and number of probiotics can be maintained stably (Guo et al., 2025).

#### 2.2.3 Bidirectional fermentation of medicinal fungi

Bidirectional fermentation of medicinal fungi is a biotechnological approach that integrates medicinal fungi (fungi used medicinally) with other biological materials, such as traditional Chinese herbal medicines, to enhance or develop active ingredients by facilitating bidirectional metabolic interactions within the same fermentation environment (Ma et al., 2025). The core of this process lies in overcoming the limitations of single medicinal fungal fermentation; the entire fermentation process is bidirectional, utilizing the exchange of substances, signal transduction, and metabolic regulation between the two subjects to produce secondary metabolites that are richer or more bioactive (Wang et al., 2024). Bidirectional fermentation of medicinal fungi represents an innovative model integrating TCM theory with modern biotechnology. By enhancing the content of active constituents, bioavailability, and pharmacological efficacy in the final products, this approach broadens the application scope of TCM while improving its safety profile. This fermentation method holds significant application prospects in the modernization of TCM, the advancement of functional foods, and the research and development of novel drugs (Wang et al., 2022).

### 2.3 Advantages of TCM fermentation

**Figure 1:**
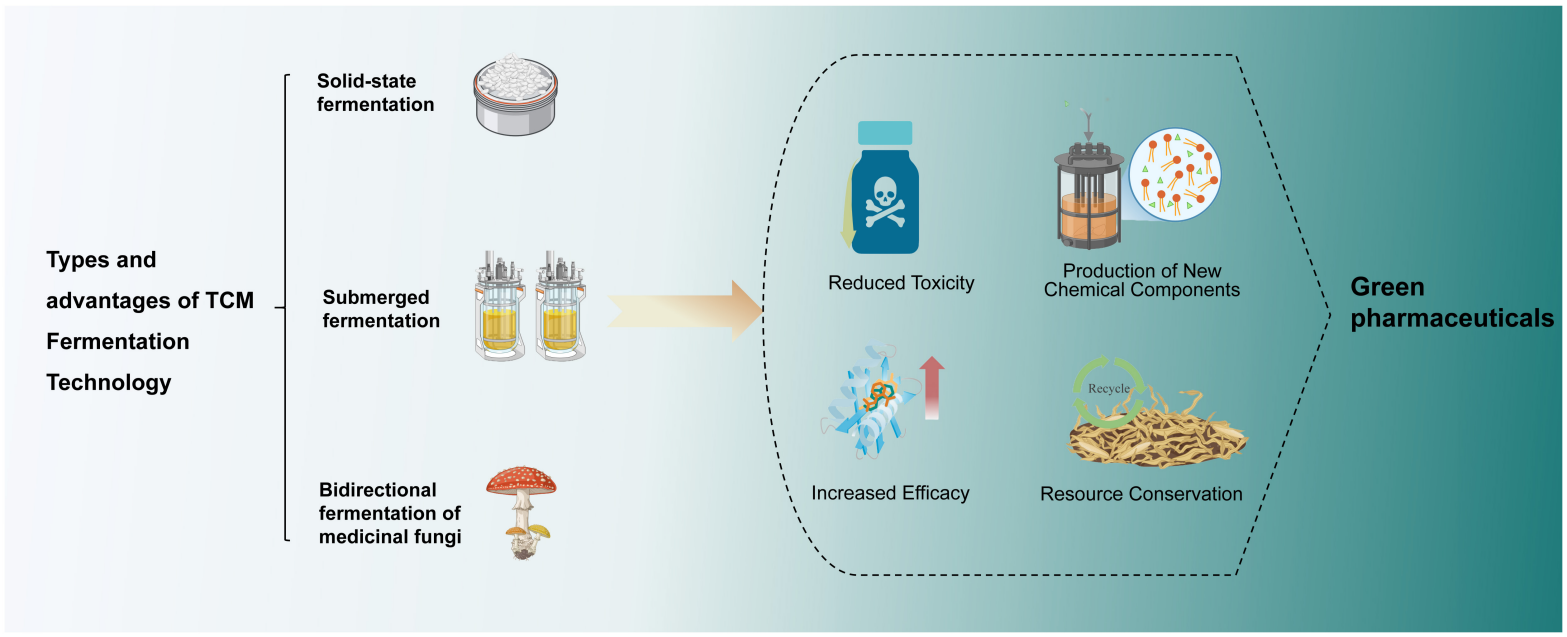
Types and advantages of TCM fermentation technology.

TCM fermentation is the process of transforming, decomposing, synthesizing, or modifying the active, inactive, or toxic components in TCM raw materials by utilizing the metabolic activities of microorganisms. Most macromolecular compounds in TCM are difficult for the body to digest, absorb and utilize without microbial fermentation. Many of the active ingredients of TCM need to be biotransformed by microorganisms in order to be biologically active (Lee et al., 2012). This also indirectly indicates that fermentation technology plays an essential role in the process of the therapeutic effectiveness of TCM. During the metabolic decomposition of substrates, microorganisms not only release primary metabolites such as ethanol and carbon dioxide, which are necessary for their growth, but also synthesize a variety of non-growth-essential compounds, which are called secondary metabolites, after the stabilization period. Their variety is extensive, covering a wide range of antibiotics, specific peptides (e.g., antimicrobial peptides), pigments, and growth factors (Méndez-Hernández et al., 2023; Robinson et al., 2001). Since these compounds have biological activities such as anti-infective, anti-inflammatory, and anticancer, they fall into the category of bioactive compounds. After fermentation treatment, TCM can improve the conversion efficiency of its intrinsic components and the rate of generation of novel compounds, making TCM fermentation technology a vital fresh approach to produce novel active compounds with strong medicinal value (Hussain et al., 2016). In addition to this, the fermentation process can effectively reduce the toxic effects of typical compounds such as lactones, toxic glycosides, and anthraquinones found in traditional Chinese medicines (Zhang X. et al., 2023). In general, TCM fermentation has the advantages of generating new substances, enhancing efficacy, and reducing toxicity. The fermentation process has the benefits of low energy consumption, simple equipment requirements, and the ability to retain more lipophilic components, thus embodying the principles of green pharmaceutical production. In recent years, the regulatory effects of fermented TCM on gut microbiota have increasingly come to public attention. This has opened up novel avenues for research in green pharmaceutical development, enabling fermentation techniques to align with TCM processing principles while simultaneously providing fresh perspectives for novel drug discovery (Figure 1).

## 3 Relationship between gut microbiota and T2DM

### 3.1 Composition and function of the gut microbiota

Initially, research proposed that the human gut microbiota consisted of around 500–1,000 bacterial species. In contrast, a recent comprehensive study revealed that the number of bacterial species in the human gut is estimated to be between 15,000 and 36,000 (Frank et al., 2007). Healthy gut microbiota consists mainly of *Firmicutes* and *Bacteroidetes*, with *Actinobacteria* and *Verrucomicrobia* as the secondary phyla (Jandhyala, 2015). These bacteria work synergistically to strengthen the intestinal epithelial barrier by regulating the expression of tight junction proteins. They also produce metabolites such as SCFAs (e.g., butyrate) that nourish intestinal mucosal cells, thereby protecting intestinal integrity and promoting overall health (Morrison and Preston, 2016). The diversity and abundance of bacteria will vary significantly with the location of the digestive tract. Notably, over two-thirds of the body's microorganisms reside in the large intestine, which predominantly comprises *Firmicutes* and *Bacteroidetes*. Conversely, species such as *Campylobacter* spp., *Salmonella* spp., *Vibrio cholerae* spp., *Escherichia coli*, and *Bacteroides fragilis* are less prevalent (Gillespie et al., 2011; The Human Microbiome Project Consortium, 2012). Typically, the gut microbiota discussed in the context of disease states refers mostly to the colonic flora. In healthy individuals, the gut microbiota maintains a symbiotic and mutually beneficial relationship with its host. This symbiotic relationship confers essential metabolic regulation, immune homeostasis, and intestinal protection to the organism (Belkaid and Hand, 2014; Sonnenburg et al., 2005). The gut microbiota functions as an “organ” within the body, performing unique roles and exerting extensive metabolic regulatory effects on the host.

### 3.2 Characterization of gut microbiota in T2DM

An increasing body of evidence indicates that the development of many chronic diseases is associated with dysbiosis of the gut microbiota (Chen Y. et al., 2021; Fan and Pedersen, 2021). The gut microbiota is a significant factor that influences the body's internal environment (Belkaid and Hand, 2014), and this association is particularly prominent in T2DM. In individuals with T2DM, the α-diversity (a measure of species richness and evenness) of the gut microbiota tends to be decreased compared to healthy individuals. Notably, an imbalance in the proportions of *Firmicutes* and *Bacteroidetes* is observed, characterized by an elevated *Firmicutes/Bacteroidetes* (F/B) ratio (Bahar-Tokman et al., 2022; Chen Z. et al., 2021). Compared with healthy individuals, both individuals with prediabetes and T2DM patients exhibit distinct differences in their gut microbiota (Zhong et al., 2019). To obtain further detailed information on the composition of the gut microbiota of patients with T2DM, Qin et al. (2012) developed a metagenome-wide association study (MGWAS) protocol. The analysis of this study revealed moderate intestinal dysbiosis in patients with T2DM, as evidenced by decreased abundance of some common butyric acid-producing bacteria and increased abundance of conditionally pathogenic bacteria. Simultaneously, microorganisms with sulfate-reducing capacity and resistance to oxidative stress were also enriched. In a study examining the gut microbiota of Indian patients with T2DM, researchers observed a characteristic pattern of microbial alterations: increased abundance of the *Firmicutes*, reduced abundance of the *Bacteroidetes*, alongside significant enrichment of the *Verrucomicrobia* and *Proteobacteria* (Beura et al., 2024). In a comprehensive review of 13 clinical trials, Wang et al. (2021) distinctly highlighted the role played by gut microbiota in the development and progression of T2DM.

### 3.3 Mechanism of gut microbiota dysbiosis leading to T2DM

**Figure 2:**
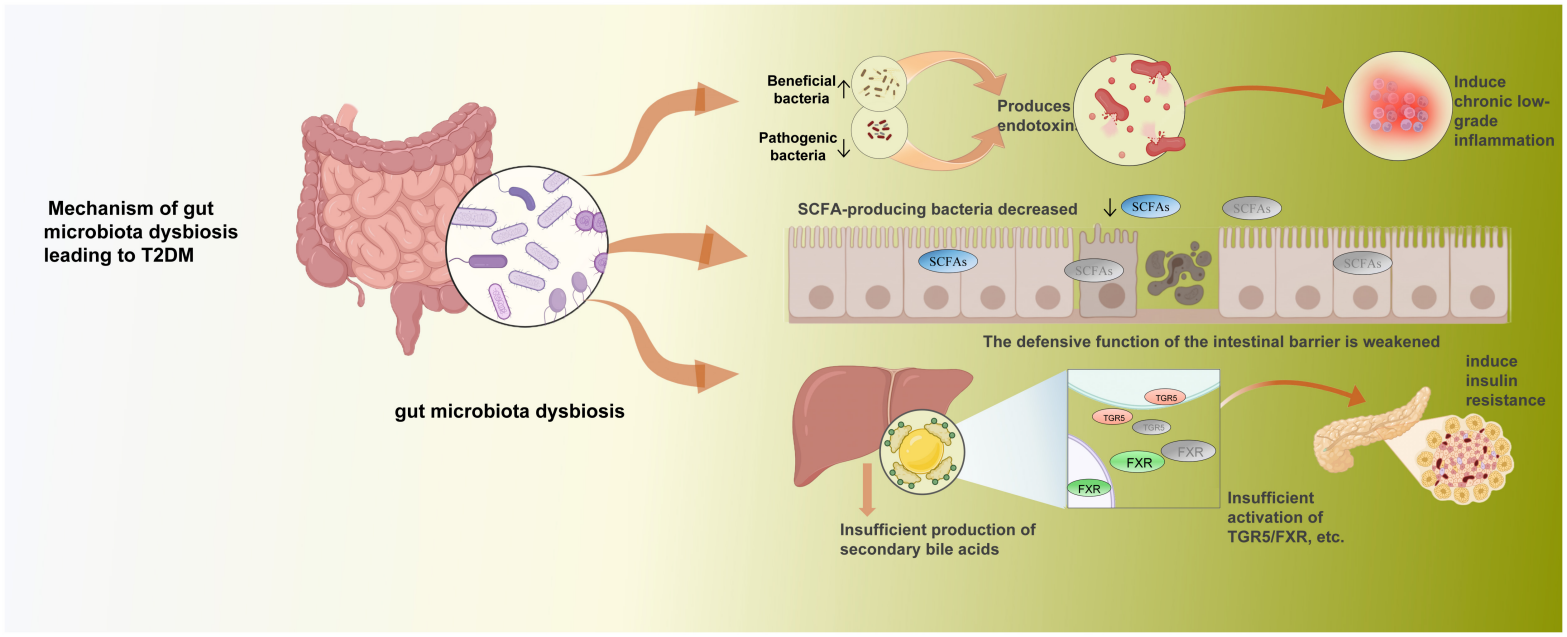
Mechanism of gut microbiota dysbiosis leading to T2DM.

The specific mechanisms whereby gut microbiota dysbiosis contributes to the development of T2DM encompass three main areas: the endotoxin theory, the short-chain fatty acid theory, and the bile acid theory (Ma et al., 2019) (Figure 2). From the endotoxin theory perspective, dysbiosis directly increases pro-inflammatory bacteria (e.g., *Escherichia coli*) while decreasing anti-inflammatory and beneficial bacteria (e.g., *Bifidobacterium*), leading to reduced microbial diversity. Opportunistic pathogens can trigger chronic low-grade inflammation by producing endotoxins (lipopolysaccharides), thereby disrupting glucose metabolism (Zhong et al., 2019; Di Vincenzo et al., 2024). Regarding the short-chain fatty acid theory, SCFAs (primarily acetate, propionate, and butyrate) are the main metabolites produced by gut microbiota fermenting dietary fiber. They serve not only as the primary energy source for intestinal epithelial cells but also effectively promote intestinal mucosal repair, enhance intestinal barrier defense functions, and play a positive preventive role in various intestinal inflammatory responses such as ulcerative colitis (Shin et al., 2023). Furthermore, substantial evidence indicates that SCFAs exert anti-obesity and anti-diabetic effects (Portincasa et al., 2022). Conversely, gut microbiota dysbiosis reduces SCFA-producing bacteria, leading to significantly diminished SCFA production. This ultimately destabilizes glucose homeostasis and induces T2DM onset (Portincasa et al., 2022; Sanna et al., 2019). In the bile acid hypothesis, bile acids function as “metabolic signaling molecules,” transmitting signals through nuclear receptors [e.g., Farnesoid X receptor (FXR)] and G protein-coupled receptors [e.g., Takeda G-protein-coupled receptor 5 (TGR5)] to regulate glucose-lipid metabolism (Singh et al., 2019). Notably, a bidirectional reciprocal relationship exists between gut microbiota and bile acids: on one hand, bile acids shape gut microbiota diversity and structural balance; on the other, gut microbiota directly determines the types and proportions of secondary bile acids (Guo et al., 2022). When gut microbiota imbalance occurs, it leads to insufficient secondary bile acid production, which in turn causes inadequate activation of TGR5/FXR. This ultimately results in insulin resistance and elevated blood glucose levels (Chen et al., 2023). More critically, insulin resistance further reduces gallbladder contractility, diminishing bile acid secretion and weakening its regulatory effect on the microbiota, thereby accelerating the progression of type 2 diabetes (Guo et al., 2022; Cadena Sandoval and Haeusler, 2025).

### 3.4 Effectiveness of regulating gut microbiota to intervene in T2DM

In recent times, a growing body of research has endeavored to enhance T2DM by regulating the gut microbiota through the supplementation of probiotics and prebiotics (Iatcu et al., 2021). Probiotics may be simply understood as a specific category of live microorganisms. When consumed in appropriate quantities, probiotics exert beneficial effects on the host's health. They can colonize directly within the gut, thereby participating in the regulation of the intestinal microecological balance. In this process, probiotics suppress the growth and reproduction of harmful bacteria by competing for nutrients and occupying living space (Wang et al., 2021). The results of several clinical studies have shown that patients with T2DM who have continuously consumed probiotics containing a variety of strains of bacteria are not only well-tolerated, but also have positive changes in a number of health indicators, such as blood glucose and blood lipids (Okesene-Gafa et al., 2020; Perraudeau et al., 2020; Zikou et al., 2023). Prebiotics (e.g., fructooligosaccharides, galactooligosaccharides, inulin, and lactulose) are non-digestible food components that selectively stimulate the growth and activity of particular beneficial bacteria (one or more species) within the gut, thereby exerting a beneficial effect on the host. Simply put, prebiotics can be analogized to “food” for beneficial bacteria (Li et al., 2021). Clinical studies have found that daily supplementation with prebiotics (i.e., inulin-type oligofructose) results in significant increases in fecal *Bifidobacterium* counts and SCFA levels in patients with T2DM. This confirms the potential of prebiotics for improving the intestinal microenvironment in patients with T2DM (Birkeland et al., 2020). It is evident that modulating the gut microbiota offers a novel strategy for improving T2DM. However, it should be noted that current research on probiotics and prebiotics still faces significant limitations, lacking unified clinical application standards. This inconsistency hinders the comparability and broader application of research findings (Suez et al., 2019; Roberfroid et al., 2010). Thus, regulating gut microbiota to improve T2DM is a new strategy. At this stage, the fermentation technology of TCM is gradually moving toward the research field, and both the pure TCM fermentation technology and the TCM combined with probiotic fermentation technology are highly respected. Compared with the traditional treatment, the advantages of TCM fermentation technology are significant. TCM fermentation technology is expected to become a novel technology for regulating the gut microbiota to assist in improving T2DM.

## 4 Research on the mechanism of TCM fermentation technology in regulating gut microbiota to improve T2DM

### 4.1 Influence of TCM fermentation products on the structure of gut microbiota

TCM fermentation technology, building upon conventional processing methods and integrating modern microbiological techniques, facilitates the decomposition and metabolism of medicinal substances. This process enhances therapeutic efficacy by expanding or generating novel active constituents. Numerous studies have shown that the bioactive components (e.g., polysaccharides and phenols) produced by TCM fermentation play a key role in regulating the gut microbiota structure, effectively increasing the abundance of beneficial bacteria in the intestinal tract, reducing the proportion of harmful bacteria, and restoring gut microbiota balance. As a special class of carbohydrates, polysaccharides derived from TCM cannot be directly digested or absorbed by the mammalian gut. In the lower part of the intestinal tract, they can play the role of “prebiotics” and become the nutrient source and substrate in the process of microbial fermentation, thus promoting the growth and activity of some beneficial bacterial communities (Wu et al., 2022). *Astragalus membranaceus* (Fisch.) Bunge, first documented in the Divine Farmer's Classic of Materia Medica, stands as a pivotal tonic herb in TCM for replenishing qi and ascending yang. It is not only frequently employed to alleviate typical qi deficiency symptoms such as fatigue and poor appetite with loose stools, but also serves as a crucial component in traditional formulations for treating “Xiaoke” (a classic TCM diagnosis characterized by polydipsia, polyphagia, polyuria and emaciation, primarily corresponding to DM in modern medicine) (Wang et al., 2023). *Paecilomyces cicadae* is a fungus with medicinal and edible properties. Zhou et al. (2022) employed SSF of *Astragalus membranaceus* (Fisch.) Bunge using *Paecilomyces cicadae*. Analysis of the SSF product revealed that flavonoids, saponins, and polysaccharides are its primary bioactive constituents. This fermentation product could effectively regulate the abundance of gut microbiota of mice with Diabetic Nephropathy (DN), including *Ruminococcaceae_UCG-014, Allobaculum, Unclassified_f__Lachnospiraceae Alloprevotella*, and *Bacteroides*. Concurrently, it improves the physiological condition of DN mice, demonstrating superior efficacy compared to *Astragalus membranaceus* (Fisch.) Bunge. Dendrobium (primarily utilizing the stem as the medicinal part) is a traditional Chinese medicinal herb renowned for its efficacy in nourishing the stomach, promoting fluid production, replenishing yin, and clearing heat. It has long been employed in the treatment of “Xiaoke.” Modern medical research has focused extensively on its rich phytochemical properties, with studies indicating its active constituents hold significant potential in both the treatment and prevention of diabetes (Li et al., 2023). Zou et al. (2025) employed fecal fluid from db/db mice to ferment *Dendrobium officinale* Kimura & Migo, *Dendrobium huoshanense* Z.Z. Tang & S.J. Cheng, *Dendrobium nobile* Lindl., and *Dendrobium chrysotoxum* Lindl., with fecal fluid from normal rats as a control. They monitored changes in the content of total polysaccharides and total polyphenols in the four Dendrobium species after fermentation. Results showed that the content of bioactive components (total polysaccharides and total polyphenols) increased in all four fermented Dendrobium species. These fermented species exhibited potent antioxidant and free radical scavenging activities, significantly regulated the diversity of gut microbiota by increasing the relative abundance of *Bacteroidota*, and promoted the production of SCFAs, thereby exerting a hypoglycaemic effect.

### 4.2 Effects of TCM fermentation technology on metabolites of gut microbiota

TCM fermentation technology can change how the gut bacteria work and impact the production of substances like SCFAs, which exert a significant impact on intestinal health and blood glucose regulation. Fermenting certain Chinese herbal medicines can boost the synthesis of SCFAs, such as acetic acid, propionic acid, and butyric acid. These metabolites play a role in adjusting intestinal pH values, participating in the regulation of energy metabolism, and delivering a beneficial effect on blood glucose management. Acetic acid primarily regulates systemic energy balance and appetite control. It enhances tissue responsiveness to insulin, promoting glucose uptake and utilization while improving insulin resistance. It also enters the central nervous system via the bloodstream, activating appetite regulation pathways to increase satiety and reduce overeating (Martin-Gallausiaux et al., 2021; González Hernández et al., 2019). Propionic acid targets glucose and lipid metabolism through hepatic metabolic pathways. Increased hepatic blood flow reduces triglyceride levels in the liver, thereby improving hepatic and systemic glucose homeostasis (Chambers et al., 2015). Butyric acid possesses potent anti-inflammatory effects, effectively strengthening tight junctions between intestinal epithelial cells. This reduces intestinal permeability, lowers systemic inflammatory responses, and alleviates insulin resistance (Wang et al., 2012). Astragalus polysaccharides (APS), as the primary constituents of the Chinese medicinal herb *Astragalus membranaceus* (Fisch.) Bunge, constitute the key active components for treating diabetes mellitus. Research has found that APS *in vitro* simulated fermentation effectively increased the abundance of beneficial bacteria in the fecal microbiota of T2DM patients, while simultaneously elevating propionic acid levels within SCFAs as measured by gas chromatography-mass spectrometry. This alteration induced GLP-1 and peptide YY (PYY) production, inhibited pancreatic β-cell apoptosis, and stimulated insulin secretion, thereby producing therapeutic improvements in T2DM (Zhang et al., 2024). *Xiexin* Decoction (XXD) is a traditional classical formula comprising three Chinese medicinal herbs: *Scutellaria baicalensis* Georgi, *Coptis chinensis* Franch., and *Rheum palmatum* L. It possesses the efficacy of clearing heat and detoxifying. Its therapeutic effect on T2DM has been validated through millennia of clinical practice, demonstrating reliable therapeutic efficacy. An experimental study investigating the effects of XXD on dyslipidemia in high-fat diet-induced obese rats revealed that this formula modulates gut microbiota composition. It promotes the fermentation of indigestible plant polysaccharides by saccharolytic bacteria in the colon, thereby increasing the production of SCFAs derived from the gut microbiome. SCFAs, by participating in energy metabolism regulation pathways, activate the peroxisome proliferator-activated receptor gamma coactivator 1-alpha (PGC-1α)/uncoupling protein-2 (UCP-2) signaling pathway. This reduces energy charge in obese rats, ultimately improving obesity-related insulin resistance (Xiao et al., 2019).

### 4.3 Protective effects of TCM fermentation technology on intestinal barrier function

An increasing number of studies have revealed that the integrity of the intestinal mucosal barrier plays a crucial role in the onset of T2DM (Shen et al., 2019). Furthermore, TCM fermentation products can improve the integrity of the intestinal mucosal barrier and reduce intestinal permeability. They also play a key role in preventing endotoxins from entering the bloodstream and alleviating the body's inflammatory response. SCFAs, as the main metabolites of gut microbiota, have been shown to be an important energy source for intestinal epithelial cells. They can modulate the activity of intestinal epithelial cells through diverse mechanisms, controlling their growth, specialization, and the activity of subsets such as enteroendocrine cells, thereby influencing intestinal movement. This also serves as a critical factor in boosting the intestinal barrier function and overseeing the host's metabolism (Zhang D. et al., 2023). While maintaining the anaerobic environment of the colon and promoting the balance of microbiota, SCFAs, particularly butyric acid, can reduce intestinal permeability and promote epithelial barrier function through the hypoxia-inducible factor (HIF) (Kelly et al., 2015). In addition, butyric acid has been found to upregulate the expression of mucin-2 (MUC2) and the goblet cell marker gene (SPDEF) in experiments conducted both in live organisms and in laboratory settings, thereby thickening the mucus layer and strengthening the protective effect of the mucous membrane (Liang et al., 2022). More importantly, the mucus layer physically blocks direct contact between endotoxins and the intestinal epithelium, reducing endotoxin adhesion and cellular invasion, thereby lowering the incidence of “leaky gut” (Di Vincenzo et al., 2024). Metabolic substances like endotoxins cannot enter the portal venous circulation, sparing the liver from inflammatory responses triggered by continuous exposure to these substances and ultimately mitigating the disruption of hepatic glucose and lipid metabolism caused by inflammation. Furthermore, when a small fraction of butyrate signals reach the liver via the portal vein, they can activate the peroxisome proliferator-activated receptor (PPAR) pathway. This pathway modulates human adipose tissue regulatory T cells, suppressing the transcription and expression of key hepatic gluconeogenesis enzymes and thereby lowering fasting blood glucose levels (Chen B. et al., 2025). Song et al. (2024a) observed that *Dendrobium officinale* Kimura & Migo extract promotes SCFA formation during *in vitro* fermentation. In db/db mice, this extract further enhances SCFA production by improving gut microbiota diversity and bolsters intestinal integrity through increased expression of colonic tight junction proteins (ZO-1 and Occludin). Furthermore, this extract may activate the PPAR pathway via the gut-liver axis, thus collectively generating advantageous impacts on metabolic syndrome triggered by T2DM.

### 4.4 Regulatory effects of TCM fermentation technology on intestinal immune function

**Figure 3:**
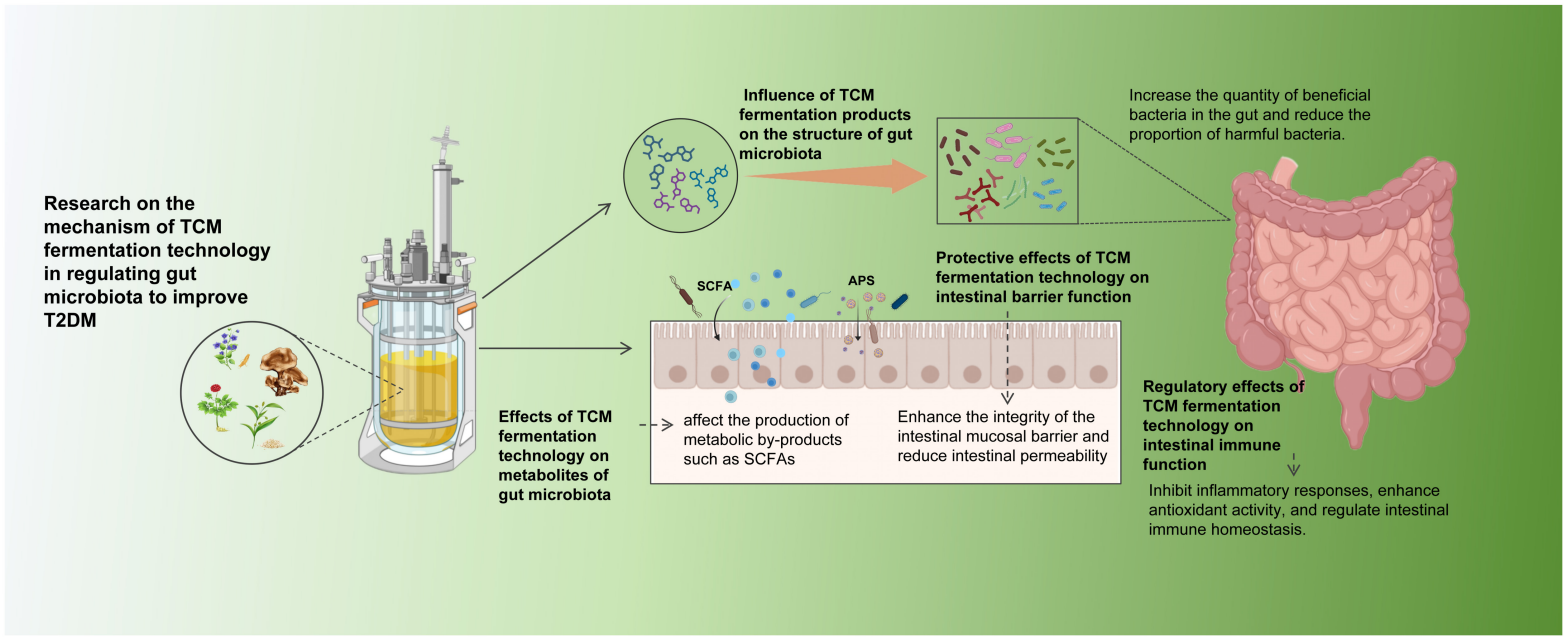
Research on the mechanism of TCM fermentation technology in regulating gut microbiota to improve T2DM.

With the advancement of research on intestinal microecology and metabolic diseases, more and more evidence suggests that intestinal immune homeostasis imbalance is an important driver of T2DM development, and that the pathological process of T2DM can be significantly ameliorated by modulating intestinal immune function (Riedel et al., 2022). Current research has definitively verified that fermented TCM, especially when fermented with *Lactobacillus*, can substantially boost its immunoregulatory efficacy. This outcome is achieved through activating the body's inherent and acquired immune mechanisms while maintaining intestinal immune balance (Zhu et al., 2022). *Ginseng trifolium* (L.) Alph.Wood, as a widely recognized medicinal plant in traditional medicine, plays an important role in guarding against and addressing T2DM (Xie et al., 2005). Fan et al. (2021) found that fermentation-treated *Ginseng trifolium* (L.) Alph.Wood could effectively alleviate lipopolysaccharide (LPS)-induced inflammatory responses and significantly enhance the structural and functional integrity of the intestinal barrier in mice by modulating the TLR4/MAPK signaling pathway. This provides a “prerequisite for restoring the integrity of the intestinal mucosal barrier and regulating intestinal immune function.” Moreover, as investigations into the metabolic derivative SCFAs have advanced, it has been unveiled that SCFAs inhibit inflammatory reactions in human monocytes by inducing the secretion of prostaglandin E2 (PGE2) and enhancing the production of the anti-inflammatory cytokine interleukin-10 (IL-10), thus preserving intestinal immune functionality (Cox et al., 2009). Zhang et al. (2024) explored the effects of APS on gut microbiota and metabolites in T2DM patients using an *in vitro* simulated fermentation model. The results showed that APS fermentation increased the levels of all-trans retinoic acid and thiamine. Both have antioxidant properties and can be enriched in KEGG pathways such as thiamine metabolism, enhancing the antioxidant properties of feces. Correlation analysis has verified a notable positive correlation between *Lactobacillus* and thiamine and DPPH clearance, indicating that the antioxidant activity of APS is linked to its capacity to enhance specific bacteria and elevate their metabolites. Modern studies have also confirmed that antioxidant activity can be regarded as a “protective shield” for intestinal immune function (Bhattacharyya et al., 2014). The antioxidant activity of APS effectively protects the integrity of the intestinal barrier and balances the function of immune cells, providing a solid foundation for intestinal immune defense. *Sijunzi* Decoction (SJZD) is a classic formula in TCM renowned for its properties of tonifying qi and strengthening the spleen. Clinically, it is frequently used as the foundational prescription for patients with T2DM who present with a spleen-deficiency syndrome, with modifications made according to individual clinical manifestations. Polysaccharides are the primary active constituents of SJZD. Using an *in vitro* simulated fermentation model, researchers have demonstrated that the purified homogeneous polysaccharide component (S-3-1) from this decoction significantly modulates the abundance of gut bacterial genera while concurrently increasing the production of SCFAs, thereby exerting immunomodulatory effects through these mechanisms (Gao et al., 2018) (Figure 3).

## 5 Application of TCM fermentation technology to improve T2DM

### 5.1 Experimental research on TCM fermentation preparations for improving T2DM

In the development of new drugs and technologies, most researchers conduct extensive and systematic experimental research to ensure the safety of subjects and guarantee the scientific validity of findings. During the experimental phase of TCM fermentation preparations for T2DM, their hypoglycaemic activity, mechanisms of action, and safety are primarily validated through cellular models, animal models, and *in vitro* simulation experiments, providing a scientific basis for subsequent clinical studies. Current research predominantly uses classical hypoglycaemic Chinese herbs as fermentation substrates, which are combined with probiotics for biotransformation. Fermentation processes are optimized to improve active ingredient content and bioavailability. The classic formula of *Danggui Buxue* Decoction (DBD) is composed of the *Astragalus membranaceus* (Fisch.) Bunge and *Angelica sinensis* (Oliv.) Diels at a 5:1 ratio. DBD is primarily valued in TCM for its efficacy in replenishing qi and generating blood. Clinically, it is frequently used to address conditions such as sallow complexion and physical fatigue, which are typical manifestations of qi-blood deficiency syndrome. This formula exerts marked therapeutic effects on prolonged cases of DM and its associated complications in patients who present with qi-blood deficiency syndrome. Guo et al. (2020) conducted relevant research in which they fermented the herbal extract of DBD. They subsequently found that the fermented DBD exhibited enhanced activities in inhibiting α-glucosidase, scavenging DPPH free radicals, and inhibiting glycosylation, resulting in effectively enhancing its antidiabetic function. *Gegen Qinlian* Decoction (GQD) is primarily used in TCM for its efficacy in resolving exterior syndromes, clearing heat, drying dampness, and relieving diarrhea. This formula exerts its effects mainly by clearing heat and drying dampness, thereby alleviating symptoms such as excessive thirst with frequent drinking, loose stools, and diarrhea. Yan et al. (2018) found that the hypoglycaemic activity of fermented GQD was significantly higher than that of unfermented GQD, and concluded that the antidiabetic activity of Chinese herbal formulas can be improved by applying fermentation technology. Red Yeast Rice (RYR) is a medicinal substance produced by fermenting rice with *Monascus purpureus*. It possesses properties that promote blood circulation and disperse blood stasis, while also fortifying the spleen and aiding digestion. It may assist in alleviating symptoms of diabetes mellitus, including fatigue and limb numbness arising from prolonged illness. Modern research has revealed its significant anti-hyperglycemic activity (Zhu et al., 2019). In a study by Chang et al. (2006), researchers administered RYR via gastric gavage for 2 consecutive weeks to diabetic rat models induced by *streptozotocin*. Results demonstrated that RYR significantly reduced plasma glucose levels in diabetic rats. Further mechanistic analysis indicated that this hypoglycaemic effect was closely associated with RYR's ability to inhibit hepatic gluconeogenesis in diabetic rats.

### 5.2 Clinical research on fermented TCM preparations for T2DM

Clinical investigations of fermented TCM preparations predominantly build upon safety and efficacy data obtained from experimental studies. Through methodologies such as randomized controlled trials (RCTs), their hypoglycaemic effects are validated in T2DM patients, aiming to explore novel approaches for clinically guarding against and addressing T2DM. Red ginseng [processed from *Ginseng trifolium* (L.) Alph.Wood], as a traditional Chinese medicinal herb, is renowned in TCM for its potent tonic properties, including greatly replenishing primordial qi (yuan qi), restoring the pulse to prevent collapse, and tonifying qi to control blood. It is frequently used to address conditions such as severe primordial qi depletion, cold limbs with a faint pulse, and fatigue arising from qi deficiency. For cases of diabetes mellitus characterized by pronounced qi deficiency, red ginseng may be incorporated to assist in the therapeutic regimen. A 4-week randomized, double-blind, placebo-controlled clinical trial conducted by the Functional Food Clinical Trial Center of Chonbuk National University Hospital of TCM found that adding fermented red ginseng (FRG) resulted in a notable decrease in postprandial blood glucose levels and raised postprandial insulin levels in comparison to the placebo group. Research findings confirmed that FRG possesses the capability to reduce postprandial blood glucose levels in subjects with impaired fasting glucose or T2DM (Oh et al., 2014). In TCM, *Chaenomeles sinensis* (Dum.Cours.) Koehne is characterized by the properties of soothing the sinews, dredging the meridians, regulating the stomach, and resolving dampness. Clinically, it is often combined with other TCM herbs to alleviate disorders of glucose and lipid metabolism in patients with T2DM. A randomized controlled clinical trial conducted at the SSRN Hospital Heart Center in Pamplemousses demonstrated that daily supplementation with 6 g of fermented *Chaenomeles sinensis* (Dum.Cours.) Koehne preparation over 14 weeks improved the overall health status of multiple organs affected by oxidative stress in patients with diabetes. These findings validate the potential application of fermented TCM in diabetes management and its associated complications (Somanah et al., 2012).

### 5.3 Application research on fermented TCM preparations modulating gut microbiota for T2DM intervention

In the field of guarding against and addressing T2DM, with the persistent rise in global incidence rates and the limitations of traditional intervention methods, finding innovative pathways that combine long-term safety, clear efficacy, and multi-target regulatory characteristics has become one of the core focuses of medical research. TCM fermentation technology, rooted in ethnic medicine, has become a focal point of research because of its distinct mechanisms of action and considerable clinical effects. It is noteworthy that, based on the preceding introduction to fermentation technology, we can observe that different fermentation processes exert a significant influence on the hypoglycemic effects of TCM. This factor has also become a critical constraint on the standardized application and maximization of efficacy for fermented TCM. Current research progress indicates that an increasing number of researchers, through experimental studies and clinical validation, have confirmed the broad application prospects of TCM fermentation technology in improving T2DM. Collectively, research findings demonstrate that the value of TCM fermentation technology in T2DM combating has been recognized by most researchers, and its translational pathway from experimental research to clinical application is becoming more evident. Based on the aforementioned exploration of mechanisms whereby fermented Chinese herbal medicines modulate gut microbiota to improve T2DM, it is reasonable to infer that among the numerous mechanisms through which fermented Chinese herbal preparations ameliorate T2DM, regulating the structure and functionality of the gut microbiota constitutes a core and pivotal component. Its significance in disease intervention has been partially substantiated by current research (Table 1).

**Table 1:** Application research on fermented TCM modulating gut microbiota for T2DM intervention.

**Fermented TCM**	**Fermentation method**	**Transformation of bioactive components/metabolites**	Influence of the gut microbiota		**Effects after fermentation**	**References**
			**Increase**	**Decrease**		
Astragalus polysaccharides [the principal constituents of *Astragalus membranaceus* (Fisch.) Bunge]	Simulated fermentation *in vitro*	SCFAs	*Dubosiella, Monoglobus*	*Escherichia-Shigella, Acinetobacter*	Stimulate GLP-1 secretion; enhance intestinal integrity	Song et al., 2024b
*Dendrobium officinale* Kimura & Migo leaf	SSF	Polyphenols	*Rikenellaceae RC9 gut group and Akkermansia*	*Lachnospiraceae UCG-001, Intestinimonas, GCA_900066575*, the value of F/B *ratio*	Lower blood sugar and regulate physiological metabolism	Chen Y. et al., 2025
*Dendrobium officinale* Kimura & Migo	Simulated fermentation *in vitro*	Total SCFA, especially acetic acid	*Dubosiella, Bifidobacterium*, and *Akkermansia*	*Escherichia-Shigella*	Lower blood sugar, regulate the immune system, and improve cognitive function	Song et al., 2025
*Zuogui Jiangtang Yishen* Decoction	Simulated fermentation *in vitro*	Tyrosine	*Parabacterioids*	*Prevotella_9*	Restore the microbiota that were altered by L-α-phosphatidylcholine and L-tyrosine to normal, along with their metabolites, improve diabetic kidney disease	Yin et al., 2025
*Achyranthes bidentata* Blume	Simulated fermentation *in vitro*	SCFAs	*Bacteroides*	*Rikenella, Alistipes, Laedolimicola, Faecalibaculum*	Promote SCFA production, alleviate diabetic kidney disease	Si et al., 2024

## 6 Prospects and challenges of TCM fermentation technology in the management of T2DM

In conclusion, TCM fermentation technology exhibits irreplaceable value in the guarding against and addressing of T2DM, with the regulation of gut microbiota as its core mechanism. Based on the regulation of gut microbiota and the improvement of T2DM, how to develop new hypoglycaemic drugs and functional foods to provide new ideas and methods for the treatment of T2DM is a challenge for us in the future. In particular, it is worth exploring how to continuously apply fermentation technology to realize the transition from fermenting single-herb medicines to TCM compounds.

However, the fermentation process is influenced by multiple factors, making precise control difficult and resulting in inconsistent quality of fermentation products. Currently, there is a lack of unified standards for the quality of fermented Chinese herbal products, and testing methods for active components vary widely. These factors collectively hinder the widespread adoption and application of such products (Yang et al., 2023). Although some studies have been conducted on the mechanisms of action, they are still not comprehensive enough, and the translation from basic research to clinical application encounters numerous challenges. Most clinical studies still have small sample sizes, short follow-up periods, and a lack of long-term safety data.

In view of the above challenges, we suggest optimizing the fermentation process parameters and utilizing advanced technology to achieve precise control of the fermentation process; formulating scientific and reasonable quality standards for the fermentation products of TCM to ensure stable and reliable product quality; further exploring the molecular mechanism of TCM fermentation technology in regulating the gut microbiota to improve the prevention and management of T2DM, and actively conducting large clinical trials to promote the practical application of TCM fermentation technology in the prevention and treatment of T2DM.

## 7 Conclusions

TCM fermentation technology can increase the utilization rate of active ingredients in TCM, reduce toxicity, or produce novel bioactive substances via microbial transformation. Additionally, it can simplify the extraction and absorption processes of medicinal ingredients, thereby enhancing the clinical applicability and safety of TCM. Fermented TCM can achieve precise improvement of T2DM by influencing the structure of gut microbiota, metabolites of gut microbiota, protecting the intestinal barrier, and regulating intestinal immune function. Therefore, by focusing on the gut microbiota, we can see that fermented TCM holds significant potential in the prevention and treatment of T2DM. Fermentation technology for TCM is poised to become a novel approach for regulating the gut microbiota and aiding in the improvement of T2DM.

## References

[B1] Bahar-Tokman H. Demirci M. Keskin F. Cagatay P. Taner Z. Ozturk-Bakar Y. . (2022). Firmicutes/bacteroidetes ratio in the gut microbiota and IL-1β, IL-6, IL-8, TLR2, TLR4, TLR5 gene expressions in type 2 diabetes. Clin. Lab. 68, 1903–1910. doi: 10.7754/Clin.Lab.2022.211244 36125161

[B2] Belkaid Y. Hand T. W. (2014). Role of the microbiota in immunity and inflammation. Cell 157, 121–141. doi: 10.1016/j.cell.2014.03.011 24679531 PMC4056765

[B3] Beura S. Kundu P. Das A. K. Ghosh A. (2024). Genome-scale community modelling elucidates the metabolic interaction in Indian type-2 diabetic gut microbiota. Sci. Rep. 14:17259. doi: 10.1038/s41598-024-63718-0 39060274 PMC11282233

[B4] Bhattacharyya A. Chattopadhyay R. Mitra S. Crowe S. E. (2014). Oxidative stress: an essential factor in the pathogenesis of gastrointestinal mucosal diseases. Physiol. Rev. 94, 329–354. doi: 10.1152/physrev.00040.2012 24692350 PMC4044300

[B5] Birkeland E. Gharagozlian S. Birkeland K. I. Valeur J. Måge I. Rud I. . (2020). Prebiotic effect of inulin-type fructans on faecal microbiota and short-chain fatty acids in type 2 diabetes: a randomised controlled trial. Eur. J. Nutr. 59, 3325–3338. doi: 10.1007/s00394-020-02282-5 32440730 PMC7501097

[B6] Cadena Sandoval M. Haeusler R. A. (2025). Bile acid metabolism in type 2 diabetes mellitus. Nat. Rev. Endocrinol. 21, 203–213. doi: 10.1038/s41574-024-01067-8 39757322 PMC12053743

[B7] Cavalieri D. McGovern P. E. Hartl D. L. Mortimer R. Polsinelli M. (2003). Evidence for *S. cerevisiae* fermentation in ancient wine. J. Mol. Evol. 57, S226–S232. doi: 10.1007/s00239-003-0031-2 15008419

[B8] Chambers E. S. Viardot A. Psichas A. Morrison D. J. Murphy K. G. Zac-Varghese S. E. K. . (2015). Effects of targeted delivery of propionate to the human colon on appetite regulation, body weight maintenance and adiposity in overweight adults. Gut 64, 1744–1754. doi: 10.1136/gutjnl-2014-307913 25500202 PMC4680171

[B9] Chang J. C. Wu M. C. Liu I. M. Cheng J. T. (2006). Plasma glucose-lowering action of Hon-Chi in streptozotocin-induced diabetic rats. Horm. Metab. Res. 38, 76–81. doi: 10.1055/s-2006-925116 16523406

[B10] Chen B. Bai Y. Tong F. Yan J. Zhang R. Zhong Y. . (2023). Glycoursodeoxycholic acid regulates bile acids level and alters gut microbiota and glycolipid metabolism to attenuate diabetes. Gut Microbes 15:2192155. doi: 10.1080/19490976.2023.2192155 36967529 PMC10054359

[B11] Chen B. Guan L. Wu C. Gong Y. Wu L. Zhang M. . (2025). Gut microbiota-butyrate-PPARγ axis modulates adipose regulatory T cell population. Adv. Sci. 12:2411086. doi: 10.1002/advs.202411086 39998325 PMC12120792

[B12] Chen Y. Yang H. Xu Z. Qu H. Liu H. (2025). *Dendrobium officinale* leaf phenolic extracts alleviate diabetes mellitus in mice via modulating metabolism and reshaping gut microbiota. J. Sci. Food Agric. 105, 5377–5387. doi: 10.1002/jsfa.14258 40156225

[B13] Chen Y. Zhou J. Wang L. (2021). Role and mechanism of gut microbiota in human disease. Front. Cell. Infect. Microbiol. 11:625913. doi: 10.3389/fcimb.2021.625913 33816335 PMC8010197

[B14] Chen Z. Radjabzadeh D. Chen L. Kurilshikov A. Kavousi M. Ahmadizar F. . (2021). Association of insulin resistance and type 2 diabetes with gut microbial diversity: a microbiome-wide analysis from population studies. JAMA Netw. Open 4:e2118811. doi: 10.1001/jamanetworkopen.2021.18811 34323983 PMC8322996

[B15] Chilakamarry C. R. Mimi Sakinah A. M. Zularisam A. W. Sirohi R. Khilji I. A. Ahmad N. . (2022). Advances in solid-state fermentation for bioconversion of agricultural wastes to value-added products: opportunities and challenges. Bioresour. Technol. 343:126065. doi: 10.1016/j.biortech.2021.126065 34624472

[B16] Cox M. A. Jackson J. Stanton M. Rojas-Triana A. Bober L. Laverty M. . (2009). Short-chain fatty acids act as antiinflammatory mediatorsby regulating prostaglandin E2 and cytokines. World J. Gastroenterol. 15:5549. doi: 10.3748/wjg.15.5549 19938193 PMC2785057

[B17] Di Vincenzo F. Del Gaudio A. Petito V. Lopetuso L. R. Scaldaferri F. (2024). Gut microbiota, intestinal permeability, and systemic inflammation: a narrative review. Intern. Emerg. Med. 19, 275–293. doi: 10.1007/s11739-023-03374-w 37505311 PMC10954893

[B18] Dilworth L. Stennett D. Facey A. Omoruyi F. Mohansingh S. Omoruyi F. O. . (2024). Diabetes and the associated complications: the role of antioxidants in diabetes therapy and care. Biomed. Pharmacother. 181:117641. doi: 10.1016/j.biopha.2024.117641 39541789

[B19] Duan Y. Guo F. Li C. Xiang D. Gong M. Yi H. . (2024). Aqueous extract of fermented *Eucommia ulmoides* leaves alleviates hyperlipidemia by maintaining gut homeostasis and modulating metabolism in high-fat diet fed rats. Phytomedicine 128:155291. doi: 10.1016/j.phymed.2023.155291 38518640

[B20] Dujic T. Zhou K. Donnelly L. A. Tavendale R. Palmer C. N. A. Pearson E. R. . (2015). Association of organic cation transporter 1 with intolerance to metformin in type 2 diabetes: a GoDARTS study. Diabetes 64, 1786–1793. doi: 10.2337/db14-1388 25510240 PMC4452716

[B21] Fan J. Liu S. Ai Z. Chen Y. Wang Y. Li Y. . (2021). Fermented ginseng attenuates lipopolysaccharide-induced inflammatory responses by activating the TLR4/MAPK signaling pathway and remediating gut barrier. Food Funct. 12, 852–861. doi: 10.1039/D0FO02404J 33404578

[B22] Fan Y. Liu Y. Shao C. Jiang C. Wu L. Xiao J. . (2025). Gut microbiota-targeted therapeutics for metabolic disorders: mechanistic insights into the synergy of probiotic-fermented herbal bioactives. Int. J. Mol. Sci. 26:5486. doi: 10.3390/ijms26125486 40564947 PMC12193472

[B23] Fan Y. Pedersen O. (2021). Gut microbiota in human metabolic health and disease. Nat. Rev. Microbiol. 19, 55–71. doi: 10.1038/s41579-020-0433-9 32887946

[B24] Frank D. N. St. Amand A. L. Feldman R. A. Boedeker E. C. Harpaz N. Pace N. R. . (2007). Molecular-phylogenetic characterization of microbial community imbalances in human inflammatory bowel diseases. Proc. Natl. Acad. Sci. U.S.A. 104, 13780–13785. doi: 10.1073/pnas.0706625104 17699621 PMC1959459

[B25] Gao B. Wang R. Peng Y. Li X. (2018). Effects of a homogeneous polysaccharide from Sijunzi decoction on human intestinal microbes and short chain fatty acids *in vitro*. J. Ethnopharmacol. 224, 465–473. doi: 10.1016/j.jep.2018.06.006 29890316

[B26] Gillespie J. J. Wattam A. R. Cammer S. A. Gabbard J. L. Shukla M. P. Dalay O. . (2011). PATRIC: the comprehensive bacterial bioinformatics resource with a focus on human pathogenic species. Infect. Immun. 79, 4286–4298. doi: 10.1128/IAI.00207-11 21896772 PMC3257917

[B27] González Hernández M. A. Canfora E. E. Jocken J. W. E. Blaak E. E. (2019). The short-chain fatty acid acetate in body weight control and insulin sensitivity. Nutrients 11:1943. doi: 10.3390/nu11081943 31426593 PMC6723943

[B28] Guo R. Guo S. Gao X. Wang H. Hu W. Duan R. . (2020). Fermentation of Danggui Buxue Tang, an ancient Chinese herbal mixture, together with *Lactobacillus plantarum* enhances the anti-diabetic functions of herbal product. Chin. Med. 15:98. doi: 10.1186/s13020-020-00379-x 32944064 PMC7488747

[B29] Guo X. Okpara E. S. Hu W. Yan C. Wang Y. Liang Q. . (2022). Interactive relationships between intestinal flora and bile acids. Int. J. Mol. Sci. 23:8343. doi: 10.3390/ijms23158343 35955473 PMC9368770

[B30] Guo X. Yu L. Liu Y. Xiao M. Zhang C. Zhao J. . (2025). Metabolic interactions between *Lactococcus lactis* and commercial starter cultures enhances the quality and flavor of fermented milk. Food Res. Int. 211:116403. doi: 10.1016/j.foodres.2025.116403 40356174

[B31] Hills R. Pontefract B. Mishcon H. Black C. Sutton S. Theberge C. . (2019). Gut microbiome: profound implications for diet and disease. Nutrients 11:1613. doi: 10.3390/nu11071613 31315227 PMC6682904

[B32] Huang P. Wang P. Xu J. Sun M. Liu X. Lin Q. . (2021). Fermented traditional Chinese medicine alters the intestinal microbiota composition of broiler chickens. Res. Vet. Sci. 135, 8–14. doi: 10.1016/j.rvsc.2020.12.021 33412475

[B33] Hussain A. Bose S. Wang J.-H. Yadav M. K. Mahajan G. B. Kim H. . (2016). Fermentation, a feasible strategy for enhancing bioactivity of herbal medicines. Food Res. Int. 81, 1–16. doi: 10.1016/j.foodres.2015.12.026

[B34] Iatcu C. O. Steen A. Covasa M. (2021). Gut microbiota and complications of type-2 diabetes. Nutrients 14:166. doi: 10.3390/nu14010166 35011044 PMC8747253

[B35] Ikusika O. O. Akinmoladun O. F. Mpendulo C. T. (2024). Enhancement of the nutritional composition and antioxidant activities of fruit pomaces and agro-industrial byproducts through solid-state fermentation for livestock nutrition: a review. Fermentation 10:227. doi: 10.3390/fermentation10050227

[B36] Jandhyala S. M. (2015). Role of the normal gut microbiota. World J. Gastroenterol. 21:8787. doi: 10.3748/wjg.v21.i29.8787 26269668 PMC4528021

[B37] Jin G. Zhao Y. Xin S. Li T. Xu Y. (2024). Solid-state fermentation engineering of traditional chinese fermented food. Foods 13:3003. doi: 10.3390/foods13183003 39335930 PMC11430836

[B38] Kato S. Sato T. Fujita H. Kawatani M. Yamada Y. (2021). Effects of GLP-1 receptor agonist on changes in the gut bacterium and the underlying mechanisms. Sci. Rep. 11:9167. doi: 10.1038/s41598-021-88612-x 33911125 PMC8080802

[B39] Kelly C. J. Zheng L. Campbell E. L. Saeedi B. Scholz C. C. Bayless A. J. . (2015). Crosstalk between microbiota-derived short-chain fatty acids and intestinal epithelial HIF augments tissue barrier function. Cell Host Microbe 17, 662–671. doi: 10.1016/j.chom.2015.03.005 25865369 PMC4433427

[B40] Krishna C. (2005). Solid-state fermentation systems—an overview. Crit. Rev. Biotechnol. 25, 1–30. doi: 10.1080/07388550590925383 15999850

[B41] Lee H. S. Kim M. R. Park Y. Park H. J. Chang U. J. Kim S. Y. . (2012). Fermenting red ginseng enhances its safety and efficacy as a novel skin care anti-aging ingredient: *in vitro* and animal study. J. Med. Food 15, 1015–1023. doi: 10.1089/jmf.2012.2187 23126662 PMC3491619

[B42] Leroy F. Charmpi C. De Vuyst L. (2023). Meat fermentation at a crossroads: where the age-old interplay of human, animal, and microbial diversity and contemporary markets meet. FEMS Microbiol. Rev. 47:fuad016. doi: 10.1093/femsre/fuad016 37076765

[B43] Li H. Y. Zhou D. D. Gan R. Y. Huang S. Y. Zhao C. N. Shang A. . (2021). Effects and mechanisms of probiotics, prebiotics, synbiotics, and postbiotics on metabolic diseases targeting gut microbiota: a narrative review. Nutrients 13:3211. doi: 10.3390/nu13093211 34579087 PMC8470858

[B44] Li J. Wang M. Liu K. Liang Y. Wang H. Lin Y. . (2025). Immunomodulatory effect of Qihuang Biwen decoction and its postbiotic product. World J. Microbiol. Biotechnol. 41:337. doi: 10.1007/s11274-025-04569-3 40999268

[B45] Li L. Wang L. Fan W. Jiang Y. Zhang C. Li J. . (2020). The application of fermentation technology in traditional chinese medicine: a review. Am. J. Chin. Med. 48, 899–921. doi: 10.1142/S0192415X20500433 32431179

[B46] Li P. Y. Li L. Wang Y. Z. (2023). Traditional uses, chemical compositions and pharmacological activities of Dendrobium: a review. J. Ethnopharmacol. 310:116382. doi: 10.1016/j.jep.2023.116382 36948262

[B47] Liang L. Liu L. Zhou W. Yang C. Mai G. Li H. . (2022). Gut microbiota-derived butyrate regulates gut mucus barrier repair by activating the macrophage/WNT/ERK signaling pathway. Clin. Sci. 136, 291–307. doi: 10.1042/CS20210778 35194640

[B48] Liu L. Zhang J. Cheng Y. Zhu M. Xiao Z. Ruan G. . (2022). Gut microbiota: a new target for T2DM prevention and treatment. Front. Endocrinol. 13:958218. doi: 10.3389/fendo.2022.958218 36034447 PMC9402911

[B49] Ma J. Wang J. Wan Y. Wang S. Jiang C. (2025). Probiotic-fermented traditional Chinese herbal medicine, a promising approach to maintaining the intestinal microecology. J. Ethnopharmacol. 337:118815. doi: 10.1016/j.jep.2024.118815 39270882

[B50] Ma Q. Li Y. Li P. Wang M. Wang J. Tang Z. . (2019). Research progress in the relationship between type 2 diabetes mellitus and intestinal flora. Biomed. Pharmacother. 117:109138. doi: 10.1016/j.biopha.2019.109138 31247468

[B51] Martin-Gallausiaux C. Marinelli L. Blottière H. M. Larraufie P. Lapaque N. (2021). SCFA: mechanisms and functional importance in the gut. Proc. Nutr. Soc. 80, 37–49. doi: 10.1017/S0029665120006916 32238208

[B52] McCreight L. J. Bailey C. J. Pearson E. R. (2016). Metformin and the gastrointestinal tract. Diabetologia 59, 426–435. doi: 10.1007/s00125-015-3844-9 26780750 PMC4742508

[B53] Méndez-Hernández J. E. Rodríguez-Durán L. V. Páez-Lerma J. B. Soto-Cruz N. O. (2023). Strategies for supplying precursors to enhance the production of secondary metabolites in solid-state fermentation. Fermentation 9:804. doi: 10.3390/fermentation9090804

[B54] Morrison D. J. Preston T. (2016). Formation of short chain fatty acids by the gut microbiota and their impact on human metabolism. Gut Microbes 7, 189–200. doi: 10.1080/19490976.2015.1134082 26963409 PMC4939913

[B55] Oh M. R. Park S. H. Kim S. Y. Back H. I. Kim M. G. Jeon J. Y. . (2014). Postprandial glucose-lowering effects of fermented red ginseng in subjects with impaired fasting glucose or type 2 diabetes: a randomized, double-blind, placebo-controlled clinical trial. BMC Complement. Altern. Med. 14:237. doi: 10.1186/1472-6882-14-237 25015735 PMC4227112

[B56] Okesene-Gafa K. A. Moore A. E. Jordan V. McCowan L. Crowther C. A. (2020). Probiotic treatment for women with gestational diabetes to improve maternal and infant health and well-being. Cochrane Database Syst. Rev. 6:CD012970. doi: 10.1002/14651858.CD012970.pub2 32575163 PMC7386668

[B57] Ong K. L. Stafford L. K. McLaughlin S. A. Boyko E. J. Vollset S. E. Smith A. E. . (2023). Global, regional, and national burden of diabetes from 1990 to 2021, with projections of prevalence to 2050: a systematic analysis for the Global Burden of Disease Study 2021. Lancet 402, 203–234. doi: 10.1016/S0140-6736(23)01301-6 37356446 PMC10364581

[B58] Perraudeau F. McMurdie P. Bullard J. Cheng A. Cutcliffe C. Deo A. . (2020). Improvements to postprandial glucose control in subjects with type 2 diabetes: a multicenter, double blind, randomized placebo-controlled trial of a novel probiotic formulation. BMJ Open Diab. Res. Care 8:e001319. doi: 10.1136/bmjdrc-2020-001319 32675291 PMC7368581

[B59] Portincasa P. Bonfrate L. Vacca M. De Angelis M. Farella I. Lanza E. . (2022). Gut microbiota and short chain fatty acids: implications in glucose homeostasis. Int. J. Mol. Sci. 23:1105. doi: 10.3390/ijms23031105 35163038 PMC8835596

[B60] Qin J. Li Y. Cai Z. Li S. Zhu J. Zhang F. . (2012). A metagenome-wide association study of gut microbiota in type 2 diabetes. Nature 490, 55–60. doi: 10.1038/nature11450 23023125

[B61] Riedel S. Pheiffer C. Johnson R. Louw J. Muller C. J. F. (2022). Intestinal barrier function and immune homeostasis are missing links in obesity and type 2 diabetes development. Front. Endocrinol. 12:833544. doi: 10.3389/fendo.2021.833544 35145486 PMC8821109

[B62] Roberfroid M. Gibson G. R. Hoyles L. McCartney A. L. Rastall R. Rowland I. . (2010). Prebiotic effects: metabolic and health benefits. Br. J. Nutr. 104, S1–S63. doi: 10.1017/S0007114510003363 20920376

[B63] Robinson T. Singh D. Nigam P. (2001). Solid-state fermentation: a promising microbial technology for secondary metabolite production. Appl. Microbiol. Biotechnol. 55, 284–289. doi: 10.1007/s002530000565 11341307

[B64] Ross F. C. Patangia D. Grimaud G. Lavelle A. Dempsey E. M. Ross R. P. . (2024). The interplay between diet and the gut microbiome: implications for health and disease. Nat. Rev. Microbiol. 22, 671–686. doi: 10.1038/s41579-024-01068-4 39009882

[B65] Sanna S. Van Zuydam N. R. Mahajan A. Kurilshikov A. Vich Vila A. Võsa U. . (2019). Causal relationships among the gut microbiome, short-chain fatty acids and metabolic diseases. Nat. Genet. 51, 600–605. doi: 10.1038/s41588-019-0350-x 30778224 PMC6441384

[B66] Shen L. Ao L. Xu H. Shi J. You D. Yu X. . (2019). Poor short-term glycemic control in patients with type 2 diabetes impairs the intestinal mucosal barrier: a prospective, single-center, observational study. BMC Endocr. Disord. 19:29. doi: 10.1186/s12902-019-0354-7 30849982 PMC6408809

[B67] Shin Y. Han S. Kwon J. Ju S. Choi T. Kang I. . (2023). Roles of short-chain fatty acids in inflammatory bowel disease. Nutrients 15:4466. doi: 10.3390/nu15204466 37892541 PMC10609902

[B68] Si H. Chen Y. Hu D. Yao S. Yang J. Wen X. . (2024). A graminan type fructan from *Achyranthes bidentata* prevents the kidney injury in diabetic mice by regulating gut microbiota. Carbohydr. Polym. 339:122275. doi: 10.1016/j.carbpol.2024.122275 38823933

[B69] Singh A. Shadangi S. Gupta P. K. Rana S. (2025). Type 2 diabetes mellitus: a comprehensive review of pathophysiology, comorbidities, and emerging therapies. Compr. Physiol. 15:e70003. doi: 10.1002/cph4.70003 39980164

[B70] Singh J. Metrani R. Shivanagoudra S. R. Jayaprakasha G. K. Patil B. S. (2019). Review on bile acids: effects of the gut microbiome, interactions with dietary fiber, and alterations in the bioaccessibility of bioactive compounds. J. Agric. Food Chem. 67, 9124–9138. doi: 10.1021/acs.jafc.8b07306 30969768

[B71] Somanah J. Aruoma O. I. Gunness T. K. Kowelssur S. Dambala V. Murad F. . (2012). Effects of a short term supplementation of a fermented papaya preparation on biomarkers of diabetes mellitus in a randomized Mauritian population. Prev. Med. 54, S90–S97. doi: 10.1016/j.ypmed.2012.01.014 22330753

[B72] Song Q. Cheng S. W. Zou J. Li K. S. L. Cheng H. Wai Lau D. T. . (2024a). Role of gut microbiota on regulation potential of *Dendrobium officinale* Kimura and Migo in metabolic syndrome: *in-vitro* fermentation screening and *in-vivo* verification in db/db mice. J. Ethnopharmacol. 321:117437. doi: 10.1016/j.jep.2023.117437 37981116

[B73] Song Q. Zou J. Cheng S. W. Li K. S. L. Lau D. T. W. Yang X. . (2025). Insights into metabolic signatures and regulatory effect of *Dendrobium officinale* polysaccharides in gut microbiota: a comparative study of healthy and diabetic status. Food Sci. Nutr. 13:e4651. doi: 10.1002/fsn3.4651 39803214 PMC11717035

[B74] Song Q. Zou J. Li D. Cheng S. W. Li K. L. S. Yang X. . (2024b). Gastrointestinal metabolism of *Astragalus membranaceus* polysaccharides and its related hypoglycemic mechanism based on gut microbial transformation. Int. J. Biol. Macromol. 280:135847. doi: 10.1016/j.ijbiomac.2024.135847 39307509

[B75] Sonnenburg J. L. Xu J. Leip D. D. Chen C.-H. Westover B. P. Weatherford J. . (2005). Glycan foraging *in vivo* by an intestine-adapted bacterial symbiont. Science 307, 1955–1959. doi: 10.1126/science.1109051 15790854

[B76] Suez J. Zmora N. Segal E. Elinav E. (2019). The pros, cons, and many unknowns of probiotics. Nat. Med. 25, 716–729. doi: 10.1038/s41591-019-0439-x 31061539

[B77] Sun L. Xie C. Wang G. Wu Y. Wu Q. Wang X. . (2018). Gut microbiota and intestinal FXR mediate the clinical benefits of metformin. Nat. Med. 24, 1919–1929. doi: 10.1038/s41591-018-0222-4 30397356 PMC6479226

[B78] The Human Microbiome Project Consortium (2012). Structure, function and diversity of the healthy human microbiome. Nature 486, 207–214. doi: 10.1038/nature11234 22699609 PMC3564958

[B79] Tian J. Li C. Dong Z. Yang Y. Xing J. Yu P. . (2023). Inactivation of the antidiabetic drug acarbose by human intestinal microbial-mediated degradation. Nat. Metab. 5, 896–909. doi: 10.1038/s42255-023-00796-w 37157031

[B80] Wang B. Wang Q. Yang Y. Zhang X. Wang J. Jia J. . (2024). Bidirectional fermentation of Monascus and Mulberry leaves enhances GABA and pigment contents: establishment of strategy, studies of bioactivity and mechanistic. Prep. Biochem. Biotechnol. 54, 73–85. doi: 10.1080/10826068.2023.2207111 37139803

[B81] Wang F. Zhao T. Wang W. Dai Q. Ma X. (2021). Will intestinal flora therapy become a new target in type-2 diabetes mellitus? A review based on 13 clinical trials. Nutr. Hosp. 39, 425–433. doi: 10.20960/nh.03866 34844413

[B82] Wang H. B. Wang P. Y. Wang X. Wan Y. L. Liu Y. C. (2012). Butyrate enhances intestinal epithelial barrier function via up-regulation of tight junction protein claudin-1 transcription. Dig. Dis. Sci. 57, 3126–3135. doi: 10.1007/s10620-012-2259-4 22684624

[B83] Wang P. Wang Z. Zhang Z. Cao H. Kong L. Ma W. . (2023). A review of the botany, phytochemistry, traditional uses, pharmacology, toxicology, and quality control of the *Astragalus memeranaceus*. Front. Pharmacol. 14:1242318. doi: 10.3389/fphar.2023.1242318 37680711 PMC10482111

[B84] Wang S. Lu L. Song T. Xu X. Yu J. Liu T. . (2022). Optimization of *Cordyceps sinensis* fermentation *Marsdenia tenacissima* process and the differences of metabolites before and after fermentation. Heliyon 8:e12586. doi: 10.1016/j.heliyon.2022.e12586 36636205 PMC9830164

[B85] Weersma R. K. Zhernakova A. Fu J. (2020). Interaction between drugs and the gut microbiome. Gut 69, 1510–1519. doi: 10.1136/gutjnl-2019-320204 32409589 PMC7398478

[B86] Wu L. Gao Y. Su Y. Li J. Ren W. C. Wang Q. H. . (2022). Probiotics with anti-type 2 diabetes mellitus properties: targets of polysaccharides from traditional Chinese medicine. Chin. J. Nat. Med. 20, 641–655. doi: 10.1016/S1875-5364(22)60210-3 36162950

[B87] Xiao S. Zhang Z. Chen M. Zou J. Jiang S. Qian D. . (2019). Xiexin Tang ameliorates dyslipidemia in high-fat diet-induced obese rats via elevating gut microbiota-derived short chain fatty acids production and adjusting energy metabolism. J. Ethnopharmacol. 241:112032. doi: 10.1016/j.jep.2019.112032 31220598

[B88] Xie J. T. Mehendale S. Yuan C. S. (2005). Ginseng and diabetes. Am. J. Chin. Med. 33, 397–404. doi: 10.1142/S0192415X05003004 16047557

[B89] Yan Y. Du C. Li Z. Zhang M. Li J. Jia J. . (2018). Comparing the antidiabetic effects and chemical profiles of raw and fermented Chinese Ge-Gen-Qin-Lian decoction by integrating untargeted metabolomics and targeted analysis. Chin. Med. 13:54. doi: 10.1186/s13020-018-0208-7 30386417 PMC6204051

[B90] Yang H. Y. Han L. Lin Y. Q. Li T. Wei Y. Zhao L. H. . (2023). Probiotic fermentation of herbal medicine: progress, challenges, and opportunities. Am. J. Chin. Med. 51, 1105–1126. doi: 10.1142/S0192415X23500519 37357176

[B91] Yin Y. Zhao C. Xiang Q. Li Z. Liu X. Hu C. . (2025). The effect of Zuogui-Jiangtang-Yishen decoction on the intestinal flora's response to L-α-phosphatidylcholine and L-tyrosine in patients with diabetic kidney disease: an *in vitro* study. Front. Pharmacol. 16:1573514. doi: 10.3389/fphar.2025.1573514 40567375 PMC12188454

[B92] Zhang D. Jian Y. P. Zhang Y. N. Li Y. Gu L. T. Sun H. H. . (2023). Short-chain fatty acids in diseases. Cell Commun. Signal. 21:212. doi: 10.1186/s12964-023-01219-9 37596634 PMC10436623

[B93] Zhang L.-X. Gao W.-Y. Wang H.-Y. (2012). [Review of traditional Chinese medicine processed by fermentation]. Zhongguo Zhong Yao Za Zhi 37, 3695–3700. 23627162

[B94] Zhang X. Jia L. Ma Q. Zhang X. Chen M. Liu F. . (2024). Astragalus polysaccharide modulates the gut microbiota and metabolites of patients with type 2 diabetes in an *in vitro* fermentation model. Nutrients 16:1698. doi: 10.3390/nu16111698 38892631 PMC11174380

[B95] Zhang X. Miao Q. Pan C. Yin J. Wang L. Qu L. . (2023). Research advances in probiotic fermentation of Chinese herbal medicines. Imeta 2:e93. doi: 10.1002/imt2.93 38868438 PMC10989925

[B96] Zheng Z. Q. Fu Q. M. Liu Y. C. (2022). Exploration of adaptation, evolution and domestication of fermentation microorganisms by applying ancient DNA technology. Yi Chuan 44, 414–423. doi: 10.16288/j.yczz.22-057 35729698

[B97] Zhong H. Ren H. Lu Y. Fang C. Hou G. Yang Z. . (2019). Distinct gut metagenomics and metaproteomics signatures in prediabetics and treatment-naïve type 2 diabetics. EBioMedicine 47, 373–383. doi: 10.1016/j.ebiom.2019.08.048 31492563 PMC6796533

[B98] Zhou Q. Yang F. Li Z. Qu Q. Zhao C. Liu X. . (2022). *Paecilomyces cicadae*-fermented Radix astragali ameliorate diabetic nephropathy in mice by modulating the gut microbiota. J. Med. Microbiol. 71, 1–12. doi: 10.1099/jmm.0.001535 35617337

[B99] Zhu B. Qi F. Wu J. Yin G. Hua J. Zhang Q. . (2019). Red yeast rice: a systematic review of the traditional uses, chemistry, pharmacology, and quality control of an important Chinese folk medicine. Front. Pharmacol. 10:1449. doi: 10.3389/fphar.2019.01449 31849687 PMC6901015

[B100] Zhu H. Guo L. Yu D. Du X. (2022). New insights into immunomodulatory properties of lactic acid bacteria fermented herbal medicines. Front. Microbiol. 13:1073922. doi: 10.3389/fmicb.2022.1073922 36519164 PMC9742447

[B101] Zikou E. Dovrolis N. Dimosthenopoulos C. Gazouli M. Makrilakis K. (2023). The effect of probiotic supplements on metabolic parameters of people with type 2 diabetes in greece—a randomized, double-blind, placebo-controlled study. Nutrients 15:4663. doi: 10.3390/nu15214663 37960315 PMC10647535

[B102] Zou J. Song Q. Lau D. T. W. Shaw P. C. Zuo Z. (2025). Gut microbiota-based bioassay for the quality evaluation of different species of Dendrobium and their therapeutic potential in type 2 diabetes. Chin. Med. 20:56. doi: 10.1186/s13020-025-01107-z 40329355 PMC12053851

