# Correction: Focus on gut microbiota regulation: exploring the potential of fermented traditional Chinese medicines in the prevention and treatment of type 2 diabetes mellitus

**DOI:** 10.3389/fmicb.2026.1821299

**Published:** 2026-03-17

**Authors:** Frontiers Production Office

**Affiliations:** Frontiers Media SA, Lausanne, Switzerland

**Keywords:** gut microbiota, novel technology, short-chain fatty acid, traditional Chinese medicine fermentation, type 2 diabetes mellitus

Senior Researcher Aleksandra P. Djukic-Vukovic was erroneously credited as a reviewer of this article.

The original version of this article has been updated.

